# Symptom relief and not cyst reduction determines treatment success in aspiration sclerotherapy of hepatic cysts

**DOI:** 10.1007/s00330-018-5851-y

**Published:** 2018-12-12

**Authors:** Myrte K. Neijenhuis, Titus F. M. Wijnands, Wietske Kievit, Maxime Ronot, Tom J. G. Gevers, Joost P. H. Drenth

**Affiliations:** 1 Department of Gastroenterology and Hepatology, Radboud University Medical Center, P.O. Box 9101, 6500 HB Nijmegen, The Netherlands; 2 Department of Health Evidence, Radboud University Medical Center, Nijmegen, The Netherlands; 3 Department of Radiology, Beaujon University Hospitals Paris Nord Val de Seine, Clichy, France

**Keywords:** Cyst, Liver, Sclerotherapy, Patient-reported outcome measures

## Abstract

**Objective:**

To assess whether quantitative assessment of symptom reduction is a better outcome parameter than cyst volume reduction for treatment success in patients treated by aspiration sclerotherapy.

**Methods:**

We included patients with symptomatic, large (> 5 cm), hepatic cysts from a randomized controlled trial (NCT02048319). At baseline and 6 months after treatment, symptoms were assessed with the polycystic liver disease questionnaire (PLD-Q) and we measured cyst volume using ultrasonography. Patient-reported change in health was assessed on a 5-point Likert scale (much worse to much better) after 6 months. We tested whether PLD-Q scores and cyst volumes changed after aspiration sclerotherapy (responsiveness). Changes in PLD-Q scores and cyst volume were compared with change in health as a measure of treatment success (discriminative ability). As secondary analysis, we compared baseline characteristics between responders (improved) and non-responders (not improved).

**Results:**

We included 32 patients. Six months after treatment, 23 patients (72%) improved. Both PLD-Q score and cyst volume significantly decreased (median 38 to 18 points, *p* < 0.001, and 479 to 68 mL, *p* < 0.001). Larger improvement in PLD-Q score was associated with a positive change in health (*p* = 0.001), while larger proportional reduction in cyst volume was not significantly associated with health improvement after treatment (*p* = 0.136). Responders had larger baseline cyst volumes compared to non-responders (median 624 mL [IQR 343–1023] vs. 322 mL [IQR 157–423] *p* = 0.008).

**Conclusion:**

Cyst diameter reduction does not reflect treatment success in aspiration sclerotherapy from patients’ perspective, while symptoms measured with the PLD-Q can be used as a reliable outcome measure.

**Key Points:**

*• Cyst diameter reduction poorly reflects treatment success in aspiration sclerotherapy.*

*• Symptoms measured by the polycystic liver disease questionnaire (PLD-Q) is a better outcome measure than cyst volume reduction for treatment success after aspiration sclerotherapy.*

*• Particularly patients with larger cysts (≥* 529 mL*) benefit from aspiration sclerotherapy.*

**Electronic supplementary material:**

The online version of this article (10.1007/s00330-018-5851-y) contains supplementary material, which is available to authorized users.

## Introduction

Hepatic cysts are fluid-filled cavities varying in volume from a few milliliters to liters [1]. Most are found incidentally as isolated cysts but some patients present with inherited polycystic liver disease (PLD) [2]. Large space-occupying cysts may cause symptoms such as abdominal pain or discomfort [3]. A minimally invasive treatment option for symptomatic large cysts is aspiration sclerotherapy, which involves complete drainage of cyst fluid followed by injection of a sclerosing agent that will eliminate the fluid-producing cyst epithelium [4].

Most studies use cyst volume reduction as a rather technical endpoint to define the efficacy of aspiration sclerotherapy [5–8]. Only a limited number of studies investigated patient-reported outcome measures such as symptomatic relief, and none of these studies used this as primary endpoint [9]. Yet, from a patient-centered view, clinical response after the procedure is the most relevant outcome as it is the patient’s independent perception of symptoms, functioning, and well-being that counts most.

Capturing the clinical response can be assessed using generic patient-related outcome measures that encompass a wide range of domains, including physical, mental, and social domains. These generic instruments such as the SF-36 lack the granularity and specificity that is needed to capture domains that are most disturbed by symptomatic hepatic cysts [10].

Recently, a validated polycystic liver disease questionnaire (PLD-Q) was developed. This disease-specific tool assesses frequency and discomfort of 13 disease-specific symptoms in PLD [11]. Symptoms arising from the presence of large simple cysts match with those in PLD [12], although it is unclear whether PLD-Q is a valuable instrument to evaluate clinical response in patients with large hepatic cysts treated with aspiration sclerotherapy.

The primary aim of this study was to assess the best outcome measure for patient-reported treatment success in aspiration sclerotherapy. Treatment success was measured by a patient-completed Likert scale for change in health as reference and compared to the PLD-Q and cyst volume reduction. As a secondary objective, we identified baseline characteristics associated with treatment success at 6 months after therapy.

## Methods

### Study population and design

We performed a post hoc analysis including patients from a previously conducted randomized, double-blind, placebo-controlled clinical trial that assessed the additional effect of the somatostatin analogue pasireotide on aspiration sclerotherapy (NCT 02048319) [8]. This study was conducted at Radboud University Medical Center, Nijmegen, the Netherlands, from April 2014 to April 2016 and approved by the Institutional Ethical Review Board. Informed consent was obtained from all patients. All patients received aspiration sclerotherapy for symptomatic, large (> 5 cm), non-neoplastic, non-hydatid hepatic cysts. Only solitary and dominant cysts (largest cyst in PLD) that were a reasonable explanation for the symptoms were included. An ultrasound-guided cyst puncture was performed for cyst fluid drainage. Subsequently, ethanol was injected and re-aspirated after 10 min of sclerotherapy. Patients were randomized (1:1) to two additional injections of pasireotide (two weeks prior and two weeks following aspiration sclerotherapy) or placebo. Endpoints included proportional change (%) in cyst volume and change in symptoms after 6-month follow-up. At 6 months, this study showed a strong reduction of the cyst volume without added beneficial effect of pasireotide (83.1% in pasireotide-arm versus 86.9% in placebo group, *p* = 0.90).

### Treatment success: change in health

In the era of patient-centered care, patients’ judgment is often used as outcome measure for treatment success [13]. Patients were asked to rate their change in health on a five-point Likert scale (much worse to much better) 6 months after treatment. We defined patients that improved (somewhat or much better) as responders, while patients that did not improve (about the same, worse, and much worse) were classified as non-responders to aspiration sclerotherapy. The use of this outcome measure for treatment success in our population was validated with two other frequently used patient-reported outcomes (see Supplementary File) [14, 15].

### Clinical outcome

To quantify changes in symptoms, patients completed the PLD-Q (version 1) on paper in person at baseline and at a follow-up visit 6 months after treatment [11]. The questionnaire included 13 symptoms that can be summed into a total PLD-Q score, ranging from 0 (asymptomatic) to 100 (severely symptomatic). Scoring and missing values were handled following the user manual [11]. For each patient, clinical response was defined as the median change in PLD-Q total score 6 months after treatment.

### Morphological outcome

**Fig. 1:**
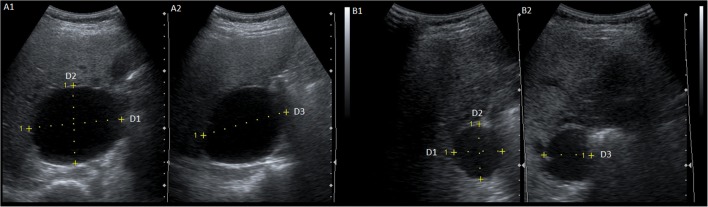
Example of ultrasound measurement of cyst volume at baseline (**A**, 220 mL) and after 6 months (**B**, 20 mL). Cyst volume was calculated by multiplying the mean orthogonal diameters using the ellipsoid formula (D1 * D2 * D3 * 0.523). In D1, we measured the cysts medio-lateral direction, D2 dorso-ventral direction, and D3 in cranio-caudal direction

Three-folded measurements of cyst volumes were performed by two blinded investigators in each patient with ultrasonography using a 3.5-MHz convex transducer (Acuson X150™, Siemens Healthineers) at baseline and 6 months after treatment. Cyst volume was calculated by multiplying the mean orthogonal diameters using the ellipsoid formula (D1 * D2 * D3 * 0.523) [16]. For each patient, morphological response was defined as the median proportional change (%) in cyst volume of the treated cyst 6 months after aspiration sclerotherapy. Figure 1 shows an example of the cyst measurements.

### Responsiveness and discriminative ability

To assess whether change in PLD-Q score is a more appropriate tool than cyst volume reduction to determine treatment success in patients with solitary cysts or PLD, we measured responsiveness and discriminative ability of these two outcome measures. Responsiveness is defined as the ability of an outcome measure to detect change over time, while discriminative ability is the degree to which a test can distinguish between groups of patients [17].

### Statistics

We pooled intervention and placebo arms for all analyzes as there was no beneficial effect of pasireotide on cyst volume reduction. Baseline characteristics were presented as absolute numbers and percentage or median and interquartile range. To assess whether we could combine patients with solitary cysts and PLD patients, we compared patient characteristics and baseline scores with Mann-Whitney *U* test or chi-square when appropriate.

We tested responsiveness of the PLD-Q and cyst volume by comparing pre- and 6 months post-treatment values with Wilcoxon signed-rank test. To assess discriminative ability, we tested whether median changes in PLD-Q scores and median proportional cyst volume reduction at 6 months were significantly different across patient-reported change in health (much worse to much better) ratings using Kruskal Wallis test. The categories “somewhat worse” and “much worse” were combined for analyses as the number of patients in these categories were very low. Thereafter, differences in change after treatment between responders and non-responders were also analyzed with Wilcoxon signed-rank test.

We compared baseline characteristics (gender, age, cyst volume, hemorrhagic cyst fluid, solitary or PLD, baseline PLD-Q score) between responders and non-responders with Mann-Whitney *U* test or chi-square when appropriate. Significant continuous factors were further explored with receiver operating characteristic (ROC) analysis to assess which cutoff point differentiated best between responders and non-responders. This cutoff point was determined by maximizing the Youden index of a ROC curve [18].

*P* values of < 0.05 were considered as statistical significant in the primary analysis. To select characteristics of response, we corrected for multiple testing using a Bonferroni correction and selected only characteristics with *p* values of < 0.0083. Analyses were performed with IBM SPSS Statistics version 22 (SPSS Inc.).

## Results

From the 34 patients included in the original trial, two patients were excluded because of failed aspiration sclerotherapy (*n* = 1) or missing baseline PLD-Q (*n* = 1). Baseline characteristics are depicted in Table 1. There were no significant baseline differences between patients with solitary cysts and PLD, enabling us to pool them.

**Table 1:** Patient demographics at baseline

	Total group *n* = 32	Solitary cysts *n* = 11	PLD *n* = 21	*p* value
Age, years	54 (48 to 61)	52 (49 to 61)	55 (47 to 61)	0.667
Female sex, *n* (%)	30 (94)	11 (100)	19 (90)	0.290
PLD-Q total score	39 (26 to 52)	44 (28 to 64)	37 (25 to 51)	0.531
Cyst volume, mL	479 (260–847)	378 (211–1253)	491 (298–728)	0.938
Hemorrhagic cyst fluid, *n* (%)	14 (44)	5 (45)	9 (43)	0.710

Variables are shown as median (IQR) unless noted otherwise. Differences between patients with solitary cysts and PLD are analyzed by Mann-Whitney *U* test or chi-square when appropriate

### Responsiveness

**Fig. 2:**
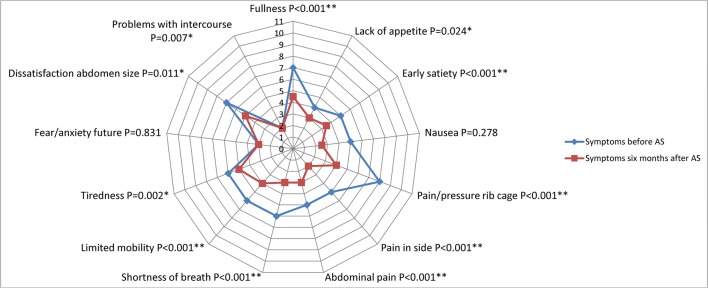
Symptoms prior to aspiration sclerotherapy (AS) compared with 6 months after therapy. *p < 0.05, **p < 0.001

Six months after aspiration sclerotherapy, there was a significant decrease in total PLD-Q score (median 38 points [26–52] to 18 points [11–33], *p* < 0.001) and cyst volume (median 479 mL [260–847] to 68 mL [11–156], *p* < 0.001). Most symptoms of the PLD-Q decreased after treatment (*p* < 0.05) (Fig. 2), except there was no significant change in nausea (*p* = 0.278) and fear or anxiety for the future (*p* = 0.831).

### Discriminative ability

As defined by patient-reported treatment success, most patients rated their change in health as improved after therapy (“somewhat better” *n* = 10, “much better” *n* = 13), six patients remained the same and health of three patients deteriorated (“somewhat worse” *n* = 1, “much worse” *n* = 2). In total, 23 patients (72%) could be defined as responder.

Table 2 shows an orderly improvement in median changes in PLD-Q total score classified by change in health (*p* = 0.001). Patient that rated themselves as “much better” decreased a median 28 points (-37 to -23), while patients that rated themselves as “worse” increased by a median 1 point (-14 to 3).

Median proportional reduction in cyst volume was not significantly different across the health status groups (*p* = 0.136). Cyst volume reduction of patients that scored themselves as “better” was similar to patients that got “worse” (median 72% [52 to 89] vs. 87% [66 to 97]).

**Table 2:** Change in health compared with change in PLD-Q score and cyst volume reduction 6 months after sclerotherapy

Self-reported rating of change	Change in PLD-Q score	Cyst volume reduction (%)
Much better, *n* = 13	-28 (-37 to -23)	72 (52 to 89)
Somewhat better, *n* = 10	-12 (-17 to -5)	90 (91 to 99)
About the same, *n* = 6	-9 (-20 to 2)	79 (60 to 97)
Worse, *n* = 3	1 (-14 to 3)^a^	87 (66 to 97)^a^

Data presented as median [IQR]

^a^Worse is a combination of the self-reported categories somewhat worse (*n* = 1) and much worse (*n* = 2)

Differences between changes in symptom scores of the PLD-Q after therapy between responders and non-responders are shown in Table 3. Responders had significant decreases in the symptoms lack of appetite, early satiety, pain in side, abdominal pain, shortness of breath, limited mobility, tiredness, dissatisfaction abdomen size, and problems with intercourse, while non-responders did not. The symptoms fullness and pain/pressure in ribcage decreased significantly in both groups.

**Table 3:** Differences in symptom scores of the PLD-Q 6 months after therapy between responders and non-responders

Symptom	Responder *n* = 23		Non-responder *n* = 11	
	Change in score	*p* value*	Change in score	*p* value
Fullness	-2 (-5 to 0)	0.002	-1 (-3 to -1)	0.040
Lack of appetite	-4 (-6 to -2)	0.042	-3 (-4 to 1)	0.330
Early satiety	-1 (-4 to 0)	0.004	-2 (-3 to 0)	0.071
Nausea	2 (2 to 4)	< 0.001	4 (2 to 4)	0.007
Pain/pressure rib cage	-4 (-5 to -2)	< 0.001	-3 (-4 to -1)	0.016
Pain in side	-3 (-6 to -2)	< 0.001	0 (-3 to 1)	0.167
Abdominal pain	-2 (-3 to -1)	< 0.001	0 (-2 to 1)	0.786
Shortness of breath	-2 (-4 to -1)	< 0.001	0 (-4 to 2)	0.497
Limited mobility	-3 (-4 to -1)	< 0.001	0 (-3 to 1)	0.750
Tiredness	-2 (-2 to 0)	0.002	0 (-2 to 1)	0.586
Fear/anxiety future	0 (-2 to 0)	0.217	1 (0 to 3)	0.102
Dissatisfaction abdomen size	-1 (-3 to 0)	0.027	-1 (-2 to 0)	0.149
Problems with intercourse	-1 (-4 to 0)	0.018	0 (-1 to 0)	0.180

Changes in PLD-Q symptoms presented as median (IQR). *p value of pre- and post-treatment scores using Wilcoxon signed-rank test

### Baseline characteristics of response

**Fig. 3:**
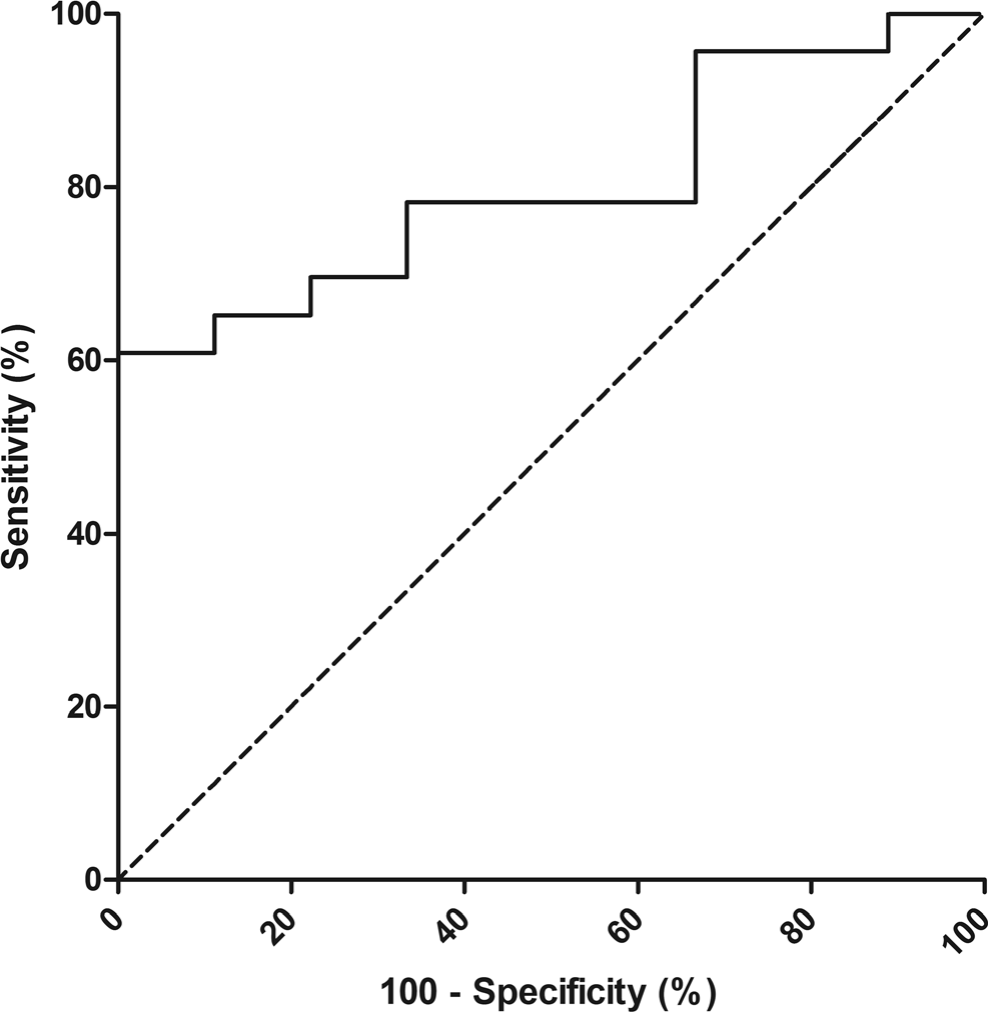
ROC analysis of baseline cyst volume and change in health. Patients were divided into two groups according to their change in health: improved (better and much better) and not improved (about the same, worse or much worse). A baseline cyst volume of ≥ 529 mL had a sensitivity and specificity of respectively 61% and 100% for improvement (AUC 0.802, *p* = 0.009)

Responders had significantly larger baseline cyst volumes compared to non-responders (median 624 mL [343–1023] vs. 322 mL [157–423] *p* = 0.008). ROC-curve analysis revealed that a baseline cyst volume larger than 529 mL was the best cutoff point to identify responders (AUC 0.802 (95% CI 0.651–0.953, *p* = 0.009; sensitivity 61%; specificity 100% (Fig. 3). Age (*p* = 0.213), gender (*p* = 0.361), solitary or PLD (*p* = 0.365), hemorrhagic cyst fluid (*p* = 0.959), and baseline PLD-Q score (*p* = 0.621) were not significantly different across responders and non-responders.

## Discussion

Our study showed that in a population of patients with large hepatic cysts, the PLD-Q and cyst volume were both highly responsive to change after aspiration sclerotherapy. We found that PLD-Q discriminated between patients who clinically benefited from this therapy or not, while cyst volume changes did not. We demonstrate that patients with larger cysts (≥ 529 mL) benefit more from aspiration sclerotherapy after 6 months than patients with smaller cysts at baseline.

The results of our study emphasize the dissociation between morphological response (cyst volume) and patient-reported outcome measures (PLD-Q) after aspiration sclerotherapy. Although larger baseline cyst volume was associated with a better outcome, dynamic changes in volume did not correlate with patient-reported treatment success. In our study, a proportion of patients reported treatment success after only a small reduction in cyst volume. This accords with previous literature indicating that one third of patients with suboptimal morphological response achieved complete clinical response [19].

A disease-specific symptom questionnaire is able to evaluate, monitor, and examine the impact of care. In addition, it can discriminate which symptoms will respond to therapy. We found that nausea did not improve after aspiration sclerotherapy. Therefore, this procedure should not be performed in case nausea is the only presenting symptom. Fullness and pain/pressure in ribcage decreased significantly in the non-responder group, but did not lead to treatment success as judged by these patients. This might suggest that these symptoms are of minor relevance to this specific patient population. Although baseline PLD-Q scores could not predict responders to treatment in our study, the PLD-Q provides quantitative response data of symptom relief and maps whether new symptoms arise (related or not to cyst of interest).

Currently, there is a large paradigm shift in medicine from morphological outcomes to patient-reported outcomes [20]. Patient-reported outcomes have gained acceptance as valid primary endpoint in studies of patients with symptomatic conditions [21]. Despite increased popularity and use of patient-reported outcome measures in radiological literature, these outcomes are still underused. Patient-reported outcomes assess the effect of trial interventions from patients’ perspectives and are particularly useful when interventions aim to improve symptoms or functional status. Our data highlight that patient-focused outcomes may be a valuable complement to the traditional clinical outcomes.

Although aspiration sclerotherapy is a highly effective procedure in terms of cyst volume reduction, 28% of patients failed to have symptomatic improvement. This highlights that a better patient selection is needed to increase its yield. Results of this study suggest that patients with a cutoff value > 529 mL, corresponding with a diameter of approximately 10 cm (calculated with ellipsoid formula), will benefit from therapy. The few available RCTs use no or a smaller cyst diameter cutoff for inclusion [7, 8, 22]. The cutoff value found in our study may be considered as evidence-based inclusion criteria for clinical trials in this field, although replication of our results is desired.

The strength of this study is that we used data of a randomized controlled trial with protocolized measurement points and an extensively validated questionnaire for polycystic liver disease. With this powerful methodology, we were able to assess which outcome measure was most appropriate to evaluate treatment success in aspiration sclerotherapy.

Limitation of this study is the small sample size. However, changes in cyst volume and symptoms were large and even using a stricter *p* value for our secondary analysis, baseline cyst volume remained a significant predictor for treatment success.

We used a questionnaire that was originally developed for polycystic liver disease. In this study, 11 (34%) patients had a solitary cyst. Based on chart review, all presenting symptoms of patients with solitary cysts were included in the PLD-Q. The PLD-Q correlated with patient-reported treatment success, confirming criterion validity, and it was responsive to therapy [17]. Although extensively assessed in cohorts of polycystic liver disease patients, reproducibility of the PLD-Q (the ability to produce the same answers in similar conditions) was not assessed in this specific population.

Moreover, 6 months of follow-up time of this study was relatively short. Possibly, patients with a clinical response and a limited cyst volume reduction may have earlier recurrence of symptoms than patients with a strong cyst volume reduction. Hypothetically, these patients may become non-responder at 12 months which may align clinical and morphological response. Longer follow-up is needed to further investigate this.

This study allows us to provide management advice on follow-up after aspiration sclerotherapy. Some centers perform routine follow-up with ultrasonography of the treated cyst after the procedure. The implication of our results is that follow-up with regular measurement of cyst size does not contribute to the magnitude of treatment success and may be thus be abolished. In case symptoms recur, cyst measurement in indicated.

In conclusion, cyst diameter reduction poorly reflects treatment success of aspiration sclerotherapy from a patient perspective, while the PLD-Q can be used as a reliable outcome measure. Our results suggest that patients with cysts > 500 mL particularly benefit from treatment.

## Electronic supplementary material

ESM 1 (DOCX 16 kb)

## Abbreviations

PLD: Polycystic liver disease
PLD-Q: Polycystic liver disease questionnaire
ROC: Receiver operating characteristic
