# Supplementary material for: Symptom relief and not cyst reduction determines treatment success in aspiration sclerotherapy of hepatic cysts
Source: Eur Radiol. 2018 Dec 12;29(6):3062–8. doi: 10.1007/s00330-018-5851-y (PMC6510865; doi:10.1007/s00330-018-5851-y)
Supplement: Supplementary file 1 — (DOCX 16 kb) [file 330_2018_5851_MOESM1_ESM.docx]

**Supplementary File 1. Validation of the outcome measure for treatment success**

***Methods***

To validate our golden standard for treatment success, we assessed whether change in cyst volume and symptoms were similarly correlated (Spearman) to change in the physical component scale of the SF-36 (score from 8-73) and the visual analogue scale for overall health (score 0-100) of the EQ5D.[[14](#_ENREF_14), [15](#_ENREF_15)]

***Results***

The PLD-Q correlated with patient-reported treatment success (r=-0.697, p<0.001), while there was no correlation with volume change (r=0.301, p=0.095). Correlations with the PCS and VAS scores revealed similar results (PLD-Q: r=-0.774, p<0.001 and r=-0.609, p<0.001 and r=0.774, p<0.001 and volume: r=-0.215, p=0.253, and r=-0.111, p=0.558), supporting the use of our golden standard as outcome for treatment success.
